# Correspondence of *D. melanogaster* and *C. elegans* developmental stages revealed by alternative splicing characteristics of conserved exons

**DOI:** 10.1186/s12864-017-3600-2

**Published:** 2017-03-16

**Authors:** Ruiqi Gao, Jingyi Jessica Li

**Affiliations:** 1 Department of Statistics, University of California, Los Angeles, USA; 2 Department of Human Genetics, University of California, Los Angeles, USA

**Keywords:** Comparative transcriptomics, Developmental stages, Conserved alternative splicing, Cassette exons, modENCODE, *D. melanogaster*, *C. elegans*

## Abstract

**Background:**

We report a statistical study to find correspondence of *D. melanogaster* and *C. elegans* developmental stages based on alternative splicing (AS) characteristics of conserved cassette exons using modENCODE RNA-seq data. We identify “*stage-associated exons*” to capture the AS characteristics of each stage and use these exons to map pairwise stages within and between the two species by an *overlap test*.

**Results:**

Within fly and worm, adjacent developmental stages are mapped to each other, i.e., a strong diagonal pattern is observed as expected, supporting the validity of our approach. Between fly and worm, two parallel mapping patterns are observed between fly early embryos to early larvae and worm life cycle, and between fly late larvae to adults and worm late embryos to adults. We also apply this approach to compare tissues and cells from fly and worm. Findings include the high similarity between fly/worm adults and fly/worm embryos, groupings of fly cell lines, and strong mappings of fly head tissues to worm late embryos and male adults. Gene ontology and KEGG enrichment analyses provide a detailed functional annotation of the identified stage-associated exons, as well as a functional explanation of the observed correspondence map between fly and worm developmental stages.

**Conclusions:**

Our results suggest that AS dynamics of the exon pairs that share similar DNA sequences are informative for finding transcriptomic similarity of biological samples. Our study is innovative in two aspects. First, to our knowledge, our study is the first comprehensive study of AS events in fly and worm developmental stages, tissues, and cells. AS events provide an alternative perspective of transcriptome dynamics, compared to gene expression events. Second, our results do not entirely rely on the information of orthologous genes. Interesting results are also observed for fly and worm cassette exon pairs with DNA sequence similarity but not in orthologous gene pairs.

**Electronic supplementary material:**

The online version of this article (doi:10.1186/s12864-017-3600-2) contains supplementary material, which is available to authorized users.

## Background

*Drosophila melanogaster* and *Caenorhabditis elegans* are two of the most intensively studied organisms in biology and serve as important model systems for investigating molecular, cellular and developmental processes of animals [1]. Separated by as many as 600 million years in evolution, *D. melanogaster* and *C. elegans* are morphologically different and evolutionarily distant organisms that have significant differences in cell differentiation and whole-organism development [2, 3]. For example, as two species in different phyla, they undertake vastly different developmental strategies, from the fixed cell lineage of *C. elegans* to the syncytial embryogenic development of *D. melanogaster* [4]. Despite these striking differences, many individual mechanisms have been observed as conserved in *D. melanogaster* and *C. elegans*, such as asymmetric cell division [5], cell migration, and axon pathfinding [6]. Additional conservation has also been observed in the regulation of *D. melanogaster* and *C. elegans* development, including the conserved temporal regulation that controls the transition from larval stages to adult stages via orthologous miRNA genes [7–9], conserved stem cell self-renewal events that are functionally important for larval and adult stages [10], and conserved cell-cell adhesion and cell-substrate adhesion molecules that are crucial to embryogenesis [4]. Moreover, conservation of transcriptome wide gene expression characteristics across developmental stages has been observed in whole-organism development of *D. melanogaster* and *C. elegans* [11]. However, there is little knowledge on the conservation of alternative splicing characteristics during the development of *D. melanogaster* and *C. elegans*, and there exists no genome-wide analysis to investigate such conservation.

Alternative splicing (AS), the process in which exons are spliced in different combinations into transcripts, is a crucial step in the regulation of vertebrate gene expression and plays an important role in the generation of proteomic diversity [12]. In evolutionary biology and comparative genomics, it is scientifically interesting and important to investigate gene regulation mechanisms by analyzing the conservation of alternative splicing across species. Between human and mouse, only 10–20% of cassette-type AS events were reported to be conserved in orthologous genes [12–15], and a similar result was found between human and rat [13]. Besides mammalian systems, low conservation of AS was also observed between model plants *Arabidopsis* and rice [16]. Although conserved AS events have low frequency in nature, studies have revealed their more important functional roles than those of species-specific AS events [17]. Examples include a higher percentage of open reading frames in conserved AS events [14, 15, 18, 19], more selection pressure on synonymous positions in codons of conserved AS exons [20], and a higher percentage of differential regulation in conserved AS events across tissues [21]. However, AS events are not static in a biological process. In the whole-organism development, they were observed to be regulated in a developmental stage specific manner [22]. Moreover, a previous study found that conserved AS events were particularly enriched in the genes involved in development [15]. These findings together suggest that it is important to study the dynamics of conserved AS events in the investigation of conserved developmental mechanisms. As the most well studied model organisms in developmental biology, *D. melanogaster* and *C. elegans* are the best targets for investigating possible conservation of AS dynamics. Existing studies have analyzed their conserved AS from several interesting perspectives, for example, a subset of conserved AS events as a possible consequence of parallel evolution [23] and similar splicing regulators of conserved AS [24]; however, there exists no study for *D. melanogaster* and *C. elegans* on their conserved AS dynamics in their development. There is also no comparison between the conservation of AS dynamics and that of gene expression dynamics.

The Model Organism ENCyclopedia of DNA Elements (modENCODE) Project [25] provides an unprecedented resource for studying genome-wide gene expression and AS characteristics in multiple *D. melanogaster* (fly) and *C. elegans* (worm) developmental stages, tissues and cells. High-throughput RNA sequencing (RNA-seq) data of 134 biological samples (including developmental stages, tissues and cells) were generated and made publicly available [26–28]. The fly time-course data contain 30 developmental stages spanning from embryos, L1–L3 larvae, pupae, to male and female adults. The worm time-course data include 36 developmental stages containing embryonic, L1–L4 larval, young adult, adult, and dauer stages. The tissue and cell data include 29 fly tissues of 10 types, 21 fly cultured cell lines, 4 worm tissues and 14 worm dissected cells (see Additional file 1: Figure S1 for more information of these biological samples; [11]). These data provide a good resource for investigating the conservation of gene expression and AS characteristics within and between fly and worm. Our previous study [11] established the first result on the correspondence of fly and worm life cycles in terms of gene expression characteristics. Two previously unknown parallel correspondence patterns were observed between fly and worm developmental stages. However, an interesting question remained unsolved: does there exist any correspondence of fly and worm life cycles in terms of AS characteristics?

**Fig. 1:**
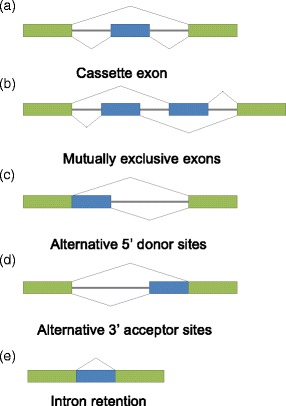
Types of alternative splicing. **a** Cassette exon; **b** Mutually exclusive exons; **c** Alternative 5’ donor sites; **d** Alternative 3’ acceptor sites; **e** Intron retention

**Fig. 2:**
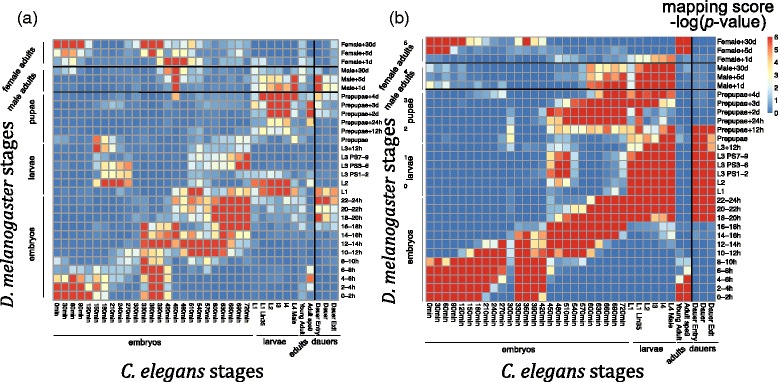
Between-species comparison of *D.melanogaster* and *C.elegans* developmental stages from different aspects. The mapping scores shown on the correspondence map are − log_10_ (*p*-value), which are calculated from the *overlap test*. **a** Mapping scores are calculated from the aspect of alternative splicing patterns. i.e., given two stages of fly and worm, the statistic is the number of conserved exon pairs in the stage-associated exons of the two species. **b** Mapping scores are calculated from the aspect of gene expression. i.e., we find stage-associated genes for every stage as the genes with high relative expression and distinguishable absolute expression at that stage. Given two stages of fly and worm, the statistic is the number of orthologous gene pairs in the stage-associated genes of the two species. Panel (**b**) has been adapted from [11]

In this study, we use these modENCODE data to compare developmental stages, tissues and cells of *D. melanogaster* and *C. elegans* based on AS characteristics of conserved exons, and compare the result with our previous result based on expression characteristics of orthologous genes [11]. We identify conserved exon pairs using criteria requiring both high DNA sequence similarity and gene orthology. We focus on fly and worm cassette exons, which constitute the most common type of alternative splicing ([29, 30]; Fig. 1). We identify “*associated exons*” to capture the AS characteristics of biological samples and use these exons to construct correspondence maps of developmental stages, tissues and cells within and between fly and worm. Within fly and worm, adjacent developmental stages are mapped to each other, i.e., a strong diagonal pattern is observed as expected, supporting the validity of our statistical approach. More importantly, the between-species mapping result reveals previously unknown correspondence of fly and worm stages in terms of AS characteristics. Interestingly, this correspondence map exhibits patterns highly consistent with the previous correspondence map we found based on gene expression characteristics (Fig. 2; [11]). This result shows that the fly and worm developmental stages with high similarity in orthologous gene expression also exhibit similar AS patterns of conserved exons. Hence, the correspondence we found between fly and worm life cycles is supported by our evidence from two different aspects: gene expression characteristics and AS characteristics. Although conserved cassette exons take up a small proportion of conserved exons, the strong patterns we find in the fly-worm correspondence maps indicate that conserved cassette exons are functionally important. We apply Gene ontology and KEGG enrichment analysis to annotate the functions of conserved cassette exons in detail. The between-species mapping results also show novel relationships of fly and worm stages, tissues and cells from the AS perspective. Moreover, we find that exon pairs with DNA sequence similarity but in non-orthologous genes are also informative for finding alternative splicing similarity of biological samples. Our study of fly and worm sample correspondence in terms of AS characteristics is innovative compared with our previous study [11] in two aspects: (1) AS events provide an alternative perspective of transcriptome dynamics, compared to gene expression events. Genes with stable expression levels but fluctuant AS levels are studied in this work but missed by our previous study, which only focused on gene expression dynamics. (2) We do not entirely rely on information of orthologous genes. We find that exon pairs with DNA sequence similarity but not in orthologous gene pairs can still lead to interesting correspondence of fly and worm samples.

## Results

### Within-species correspondence maps of *D. melanogaster* developmental stages and tissues/cells based on alternative splicing characteristics

**Fig. 3:**
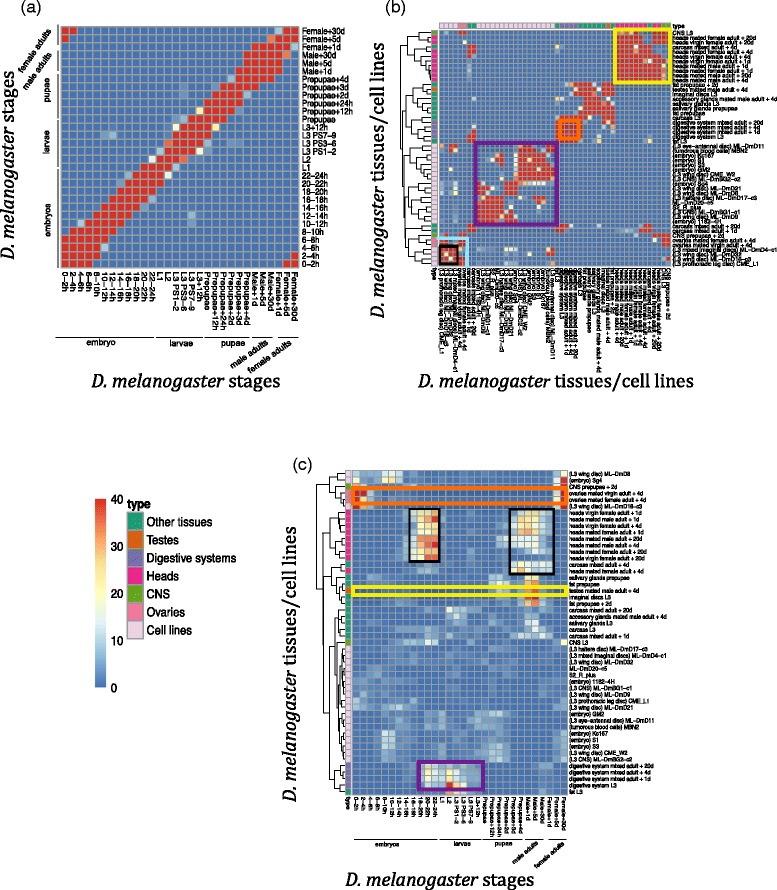
Comparison results of developmental stages, tissues and cell lines based on conserved AS dynamics within fly. The mapping scores shown on the correspondence map are − log_10_ (*p*-value), which are calculated from the *overlap test*. **a** Comparison of developmental stages. **b** Comparison of tissues/cell lines. **c** Comparison of developmental stages with tissues/cell lines. Hierarchical clustering is applied to order tissues/cell lines in (**b**) and (**c**). Tissue and cell types are labelled with colors

We first apply our strategy to comparing developmental stages, tissues and cultured cell lines within *D. melanogaster*. The correspondence map of 30 fly developmental stages is shown in Fig. 3a. As expected, there is a strong diagonal pattern in the map, indicating that adjacent fly developmental stages close to each other in time order are mapped to each other. We also observe several off-diagonal mappings, including mappings (1) between early embryos and female adults, (2) between middle embryos and larvae. The first mapping between early embryos (i.e., embryo 0–4 h) and female adults (i.e., female 5-30d) agrees with the observation of an independent study [31] and can be explained by the expression of maternal effect genes. The second mapping between middle embryos (i.e., embryo 10–14 h) and larvae (i.e., L3 PS7-9) is consistent with previous microarray profiling analysis [32]. Moreover, all these mappings agree with our previous result based on gene expression characteristics [11]. Together, the within-stage mapping results support the validity of our approach.

Figure 3b summarizes the mapping results of 29 fly tissues and 21 fly cultured cell lines (ordered by hierarchical clustering). Cell lines form two strong groupings with each other (black box and purple box), which are separated from their originating tissues, suggesting that cultured cell lines share similar transcriptome characteristics not found in tissues. This pattern is consistent with what we observed in terms of gene expression [11]. However, compared to gene expression results, cell lines are divided into smaller blocks, indicating that cell lines have greater AS diversity than gene expression diversity. Ovaries are grouped with a few cell lines (between the lower left black box and the cyan lines), due to AS characteristics of exons in maternal effect genes. It is also consistent with the reported similarity between cell lines and early embryos [31], the gene expression mapping results [11] and our observed mapping of early embryos to female adults (Fig. 3a). In addition, different head and CNS tissues (i.e., heads of virgin female, mated male and mate female adults +1, 4 and 20d, CNS L3) are mapped to each other (yellow box) and so are the digestive systems (i.e., digestive system mixed adult + 1, 4, 20d, orange box), indicating strong similarity of tissues of the same type regardless of stage or sex.

Next we compare the 50 fly tissues and cultured cell lines with the 30 fly developmental stages by the same approach (tissue/cells ordered by hierarchical clustering). From the results shown in Fig. 3c, we observe two patterns consistent with gene expression mapping results [11]: 1) ovary tissues mapped to both embryos and female adults, which again confirms that highly included or skipped exons in maternal effect genes result in the strong correspondence of fly early embryos and female adults (orange box); 2) testis tissues mapped to fly male adults (yellow box). Interestingly, head tissues are grouped together and mapped to both late embryos and the stages from prepupae to male adults (two black boxes), suggesting that fly may undergo fast head development during these two periods. In addition, digestive systems are mapped to the stages from late embryos to larvae (purple box), indicating the development of digestive systems during those stages. These mapping results (i.e., stages with stages, tissues/cells with tissues/cells and tissues/cells with stages) together provide new knowledge on the similarity of biological samples within fly from the perspective of AS characteristics.

### Within-species correspondence maps of *C. elegans* developmental stages and tissues/cells based on alternative splicing characteristics

**Fig. 4:**
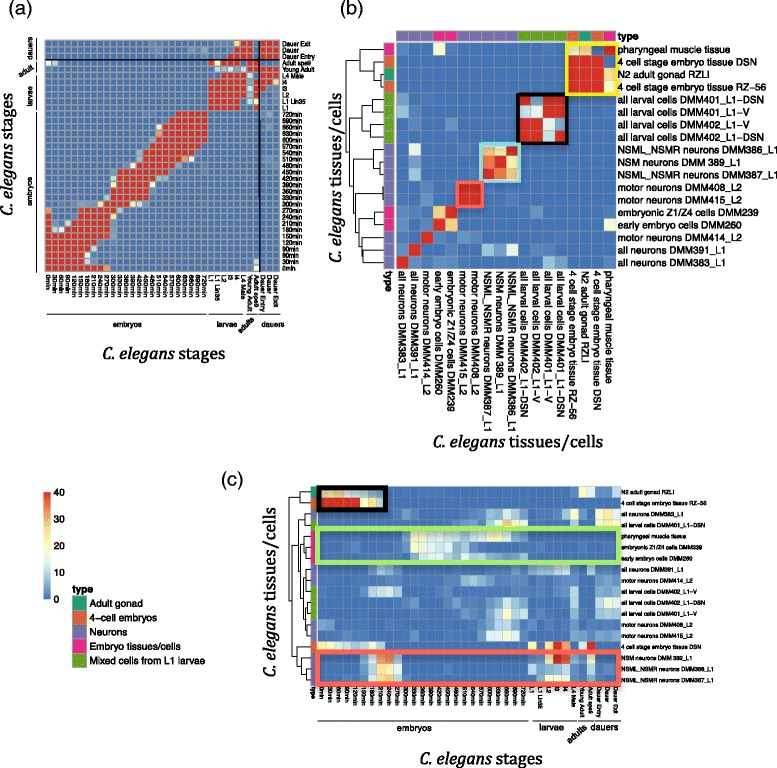
Comparison results of developmental stages, tissues and dissected cells based on conserved AS dynamics within worm. The mapping scores shown on the correspondence map are − log_10_ (*p*-value), which are calculated from the *overlap test*. **a** Comparison of developmental stages. **b** Comparison of tissues/cell lines. **c** Comparison of developmental stages with tissues/dissected cells. Hierarchical clustering is applied to order tissues/dissected cells in (**b**) and (**c**). Tissue and cell types are labelled with colors

We next apply the same strategy to comparing developmental stages, tissues and cultured cell lines within *C. elegans*. From the resulting mapping of 36 worm developmental stages in Fig. 4a, we observe a strong diagonal pattern as expected. There are also several off-diagonal mappings, the clearest of which is between worm early embryos (i.e., embryo 0–30 min) and adults (i.e., adult *spe9*). Since worms are ~99.5% hermaphrodites that produce their sperm in the L4 stage and then turn to producing oocytes in adults [33], the observed mapping is probably due to maternal gene regulation in worm oocytes.

Figure 4b summarizes the mapping results of 4 worm tissues and 14 dissected cells (ordered by hierarchical clustering). Unlike fly tissues/cells mapping, worm tissues and cells from similar origins show strong groupings with each other: 1) cells dissected from L1 stage-worms are grouped together (black box); 2) dissected neuron cells are mapped to each other, with higher similarity among the neuron cells of similar types (cyan and orange boxes). In addition, we observe that the 4-cell stage tissues and the embryonic muscle tissue (i.e., pharyngeal muscle tissue) are mapped to the adult gonad tissue (yellow box), further indicating that our mapping results of early embryos to adults in Fig. 4a are likely attributed to the AS characteristics of maternal effect genes in gonad tissues and early embryos.

Next we compare the 18 worm tissues and dissected cells with the 36 worm developmental stages. The resulting correspondence map is shown in Fig. 4c. Several informative and reasonable patterns are shown in this map. First, embryonic tissue/cells are mapped to late embryonic stages (green box), which is consistent with previous results based on gene expression [11]. Second, adult gonad tissues are mapped to early embryos together with the 4-cell embryonic tissues (black box), again supporting the important role of AS characteristics of maternal effect genes in gonad tissues. Third, certain types of neuron cells (i.e., NSM, NSML and NSMR neuron cells) are mapped to early-middle embryos and the stages from larvae to adults, indicating that fast neuron development of worm may take place during these two periods.

### Between-species correspondence maps of *D. melanogaster* and *C. elegans* developmental stages and tissues/cells based on alternative splicing characteristics

We next apply our strategy to comparing developmental stages, tissues and cells between fly and worm. As the first attempt to compare the life cycles of *D. melanogaster* and *C. elegans* from the perspective of alternative splicing characteristics, we first compare 30 fly developmental stages with 36 worm developmental stages, and illustrate the resulting mapping score matrix of 30 × 36 dimension as a correspondence map (Fig. 2a). A larger mapping score indicates a stronger association of the corresponding fly and worm stages in terms of conserved alternative splicing. Our results find two parallel patterns in the correspondence map, with a division of the two patterns in the middle of the fly life cycle: 1) the first half of the fly life cycle, from early embryos and early larvae, are well aligned to the worm life cycle; 2) fly late larvae, prepupae, and male adults are mapped to worm late embryos, larvae, and adults, respectively. Fly male adult stages are also mapped to worm dauer stages, but there is no such correspondence between fly female adult stages and worm adults or dauers. Besides the two parallel patterns, we also see that worm early and middle embryos are mapped to fly female adults, and worm middle embryos are also mapped to fly larvae.

Interestingly, this correspondence map is very similar to a previous correspondence map we found between fly and worm life cycles based on gene expression characteristics (Fig. 2b). In next section, we will compare the two correspondence maps and explain their similarities and differences.

**Fig. 5:**
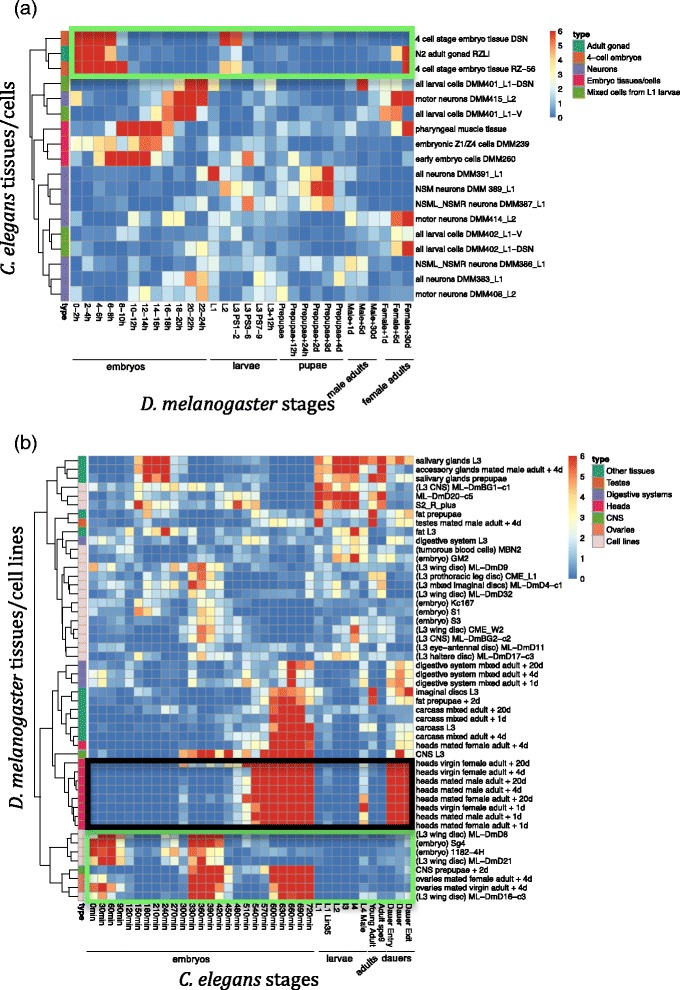
Comparison results of developmental stages, tissues, cell lines and dissected cells based on conserved AS dynamics between fly and worm. The mapping scores shown on the correspondence map are − log_10_ (*p*-value), which are calculated from the *overlap test*. **a** Comparison of fly developmental stages with worm tissues/dissected cells. **b** Comparison of fly tissues/cell lines with worm developmental stages. Hierarchical clustering is applied to order fly tissues/cell lines and worm tissues/dissected cells in (**a**) and (**b**). Tissue and cell types are labelled with colors

In order to measure the similarity of AS patterns of tissues/cells and developmental stages between two species, we use the same between-species comparison strategy to compare 1) 18 worm tissues/cells with 30 fly developmental stages (Fig. 5a) and 2) 50 fly tissues/cells with 36 worm developmental stages (Fig. 5b). In Fig. 5a, worm 4 cell stage tissues and adult gonad tissues are mapped to both fly early embryos and female adults (green box); in Fig. 5b, fly ovary tissues and several embryo cells are mapped to worm early embryos and late embryos (green box). This two results are consistent with mapping results based on gene expression [11], and confirm the strong conservation of fly and worm exons in maternal oocyte genes and their AS characteristics, which are also observed in Fig. 2a. In addition, Fig. 5b shows that fly head tissues are mapped to worm late embryos and dauers (black box). Combining this mapping result with the within-fly result in Fig. 3c that shows fly head tissues mapped to fly late embryos and male adults, as well as the between-species result (Fig. 2a) that worm late embryos and dauers are mapped to fly late embryos and male adults, we reach a conclusion that AS characteristics of fly head tissues play a major role in the between-species comparison.

### Comparison of the fly-worm stage correspondence maps based on alternative splicing characteristics or gene expression characteristics of developmental stages

In our previous study [11], we found a correspondence map with similar patterns based on gene expression characteristics of stages. Specifically, we found stage-associated genes for every stage as the genes with high relative expression and distinguishable absolute expression at that stage. Subsequently we compared a fly and worm stage pair by using a similar overlap test on the number of orthologous gene pairs in their stage-associated genes. Interestingly, the two correspondence maps we found from different aspects (one based on AS characteristics (Fig. 2a) and the other based on gene expression characteristics (Fig. 2b)) have great similarity. First, they both have two parallel patterns that are divided in the middle of fly developmental stages. Second, they share important mapping patterns, including the mapping of worm early embryos with fly female adults (in the top left corners of Fig. 2a-b), and the mapping of worm adult *spe9* stage with fly early embryos (in the bottom right of Fig. 2a-b).

Despite these major agreements between the two correspondence maps, there exist slight differences between them. Such differences indicate the different roles of AS and gene expression characteristics in fly and worm development. There are three unique patterns only existing in the AS correspondence map (Fig. 2a): the mapping of worm dauers with fly male adults, the mapping of worm late embryos with fly male adults and the mapping of worm early embryos with fly larvae. In the gene expression correspondence map (Fig. 2b), there is also a unique mapping of worm adults with fly female adults. Another difference is that the upper parallel pattern in the AS correspondence map is slightly lower than in the gene expression correspondence map. In other words, the same worm stages (from late embryos to adults *spe9*) are aligned to earlier fly stages based on AS. This result implies that conserved AS may precede conserved gene expression after the morphogenesis in fly development. This is a reasonable hypothesis, as AS is a factor that affects the overall gene expression.

### Analysis of the fly-worm stage correspondence maps based on AS characteristics encoded in different sets of exon pairs

**Fig. 6:**
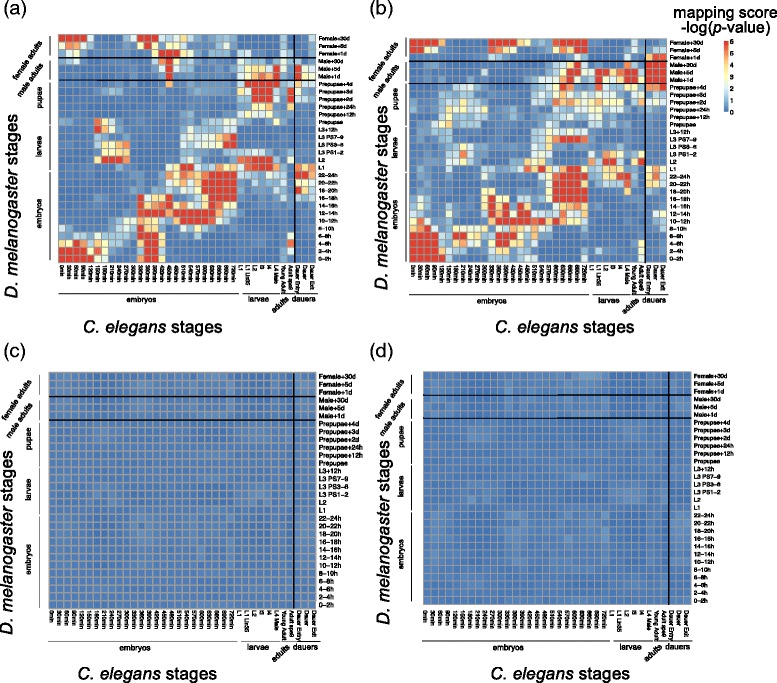
Between-species alternative splicing patterns comparison restricted to two categories of exon pairs: **a** conserved exon pairs. **b** exon pairs that are not from an orthologous gene pair but have high DNA sequence similarity. **c** mean correspondence map of negative controls: shuffle worm cassette exons in (**b**) 100 times and pair them with fly cassette exons in (**b**). **d** standard deviation of correspondence maps of negative controls

We would like to further explore if AS characteristics of the exon pairs that have high DNA sequence similarity can provide similar or even additional information than those of the conserved exon pairs to the comparison of fly and worm developmental stages. Before this exploration, we first define two sets of exon pairs: (a) 2148 conserved exon pairs, whose every pair is from an orthologous gene pair and has high DNA sequence similarity (i.e. E-value < 10-5 by BLAST) and (b) 3179 cassette exon pairs that have high DNA sequence similarity (i.e. E-value < 10^-5^ by BLAST) and are not from an orthologous gene pair. Then we apply the same comparison strategy to the exon pairs in each of the two sets (Fig. 6). The resulting two correspondence maps have overall similar patterns, but with slight differences in mapping and noise levels. The clearer pattern in Fig. 6a (same as Fig. 2a) comes from the conserved exon pairs (i.e., set (a)). This result shows that the conserved AS dynamics in orthologous genes are informative. Nevertheless, the exon pairs in set (b), which are not from an orthologous gene pair, can also lead to an informative correspondence map (Fig. 6b) that contains several interesting patterns different from Fig. 6a, including 1) clearer mapping of worm dauers with fly male adults; 2) unique mapping of worm late embryos with fly late prepupaes and male adults. These results indicate that cassette exons with similar DNA sequences but in non-orthologous genes may also share similar AS characteristics in fly and worm development. To verify that these results are not random phenomena, we conduct a negative control experiment, where in set (b) we randomly shuffle worm cassette exons and pair them with fly cassette exons to create “pseudo” cassette exon pairs. Then we apply the same algorithm to these “pseudo” cassette exon pairs. We repeatedly conduct this experiment for 100 times and report the mean and standard deviation of the correspondence map in Fig. 6c and d respectively. The resulting mean of correspondence map shows no interesting patterns, and different stage pairs exhibit similar standard deviations, indicating that the patterns we observe in Fig. 6b indeed attribute to the DNA sequence similarity of exon pairs. We also analyze the gene ontology (GO) terms of the genes corresponding to the exon pairs in set (b). The following 4 biological process GO terms are shared by more than 100 exon pairs: ATP binding, protein phosphorylation, integral component of membrane, and transmembrane transport. This result indicates that cassette exons with similar DNA sequences but not in orthologous genes might play similar roles in basic cellular and biochemical processes.

### Gene ontology/Kyoto Encyclopedia of Genes and Genomes enrichment analysis

**Fig. 7:**
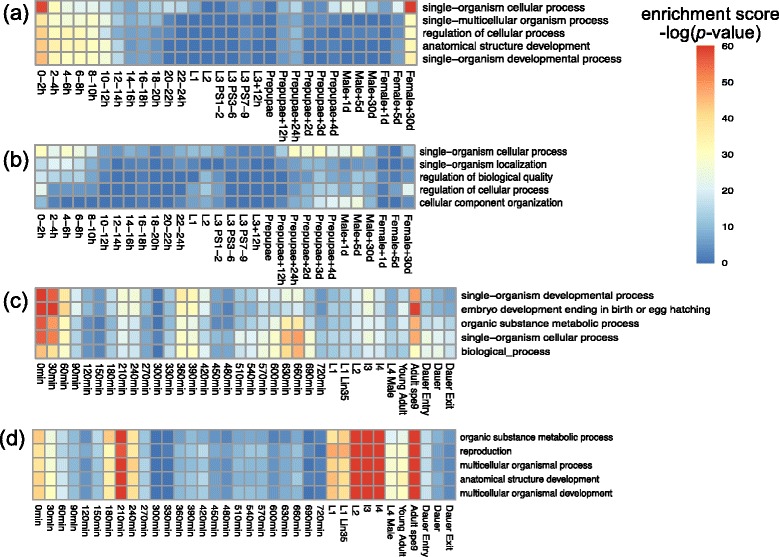
Top 5 enriched GO terms of (**a**) fly highly included associated exons, (**b**) fly lowly included associated exons, (**c**) worm highly included associated exons and (**d**) worm lowly included associated exons

To understand our results from a functional perspective, we calculate the enrichment of biological process (BP) gene ontology (GO) terms of the identified stage-associated exons (See Methods for the definition of associated exons) by a hypergeometric test. Specifically, for a given GO term, we compare the proportion of genes that contain stage-associated exons and have this GO term annotation, to the proportion of genes with this annotation in the whole genome. If the former proportion is significantly higher, we regard this particular GO term to be enriched in the stage-associated exons. We summarize the top enriched GO terms (i.e., *p*-value of the hypergeometric test < 10^-30^) in the highly-included and lowly-included stage-associated exons of fly and worm developmental stages in Additional file 2. We list the top 5 enriched GO terms (across all fly/worm stages) whose minimum depth from root in the gene ontology graph are 3 in Fig. 7. Interestingly, we observe that GO terms related to single-organism processes are enriched in the highly included stage-associated exons of both fly and worm embryos, fly female adults, and worm adults (Fig. 7a). This result again confirms the observed mapping of fly and worm embryonic and adult stages. Moreover, the top enriched GO terms in worm highly included and lowly included stage-associated exons differ greatly (Fig. 7b). Terms related to single-organism development are enriched in highly included associated exons in worm embryonic and adult stages, while terms related to multicellular development are enriched in those lowly included associated exons in worm larva and adult stages.

Kyoto Encyclopedia of Genes and Genomes (KEGG) is a database resource for understanding high-level functions and utilities of the biological system. We also calculate the enrichment of KEGG terms in the identified stage-associated exons using the same approach as for the GO enrichment analysis. The enrichment results are summarized in Additional file 3. As expected, “spliceosome” is the most highly enriched term in fly highly included stage-associated exons. Many other basic molecular pathways, such as “citrate cycle (TCA cycle)”, “glycolysis”, and “pyruvate metabolism”, are enriched in both fly and worm stage-associated exons, implying that AS dynamics have regulatory roles in these pathways.

### Functional annotation of protein domains encoded by conserved cassette exons

To further understand the functions of conserved cassette exons, we annotate the functions of protein domains encoded by conserved cassette exons using the InterProScan software package [34]. Specifically, we translate the open reading frames of cassette exons into protein sequences and use InterProScan to scan the protein sequences against InterPro’s signatures. 1032 fly exons and 1201 worm exons have been annotated. Among 2148 conserved exon pairs, fly and worm exons in 1215 pairs each share the same functional annotations. We summarize the shared functional annotations of protein domains encoded by conserved exon pairs in Additional file 4. We also list the top 10 most frequent functions among the functions encoded by the 1215 exon pairs in Table 1, and we find it very interesting that many of these conserved cassette exon pairs encode protein domains responsible for binding processes, catalytic activities, and membrane biology, which are all key regulatory processes at the molecular and cellular levels. Given that we observe these exon pairs have similar AS dynamics in fly and worm development, it is reasonable that they encode important protein domains for molecular cell regulation. These conserved cassette exons and their corresponding protein domains may serve as important targets for further understanding of the conservation of developmental biology.

**Table 1:** Top 10 most frequent functions of protein domains encoded by conserved exon pairs

Function	# Occurrences	Function	# Occurrences
ATP binding	292	Protein kinase activity	80
Protein binding	109	Protein phosphorylation	79
Integral component of membrane	105	Nucleotide binding	72
Catalytic activity	89	Metabolic process	70
Oxidation-reduction process	86	Membrane	68

## Discussion

In this paper, we explore the conservation of *D. melanogaster* and *C. elegans* from a novel aspect: alternative splicing characteristics. Our approach focuses on identifying associated-exons based on inclusion/skipping ratios of cassette exons across biological samples, and statistically testing the dependence of two biological samples based on stage-associated exons. Using this approach, we provide a comprehensive comparison of developmental stages, tissues and cells within and between *D. melanogaster* and *C. elegans*. Our comparison results reveal previously unknown mappings of stages, tissues and cells both within and between the two species from the perspective of AS. More importantly, we find two parallel correspondence patterns between fly and worm developmental stages. Although the correspondence map based on AS characteristics is overall similar to the other correspondence map we previously found based on gene expression characteristics, we observe new correspondence patterns between certain fly and worm stages only supported by AS characteristics.

We further investigate what information is provided by the exon pairs with high DNA sequence similarity in addition to conserved exon pairs in the establishment of the fly-worm stage correspondence map. Our results show that the AS characteristics of the exon pairs not in any orthologous gene pairs can still lead to a clear correspondence map. This implies that conserved AS at the exon level in non-orthologous genes can still be biologically meaningful and functionally important. A further implication of our results is the cooperativity of transcription and post-transcriptional control in conserved biological processes.

Our approach is directly applicable as a general method to compare biological samples in terms of alternative splicing patterns. It can also be used to explore other types of alternative splicing patterns (i.e. mutually exclusion, alternative 5’, alternative 3’ and intron retention). The stage-associated exons identified by our approach can provide further biological insights into the conservation of fly and worm developmental biology. For example, the conserved exons that are associated with both fly and worm embryos are possibly important functional elements in conserved embryonic developmental programs. Moreover, the comparison of stage-associated exons and stage-associated genes provides an interesting future direction to explore the relationship of alternative splicing and gene expression in fly and worm development. For example, a gene that is associated with a later stage and whose cassette exon is associated with an earlier stage is a potential target for investigating splicing regulated gene expression in developmental programs. Also, it is worthwhile to study how these conserved alternative spliced genes affect other orthologous but not alternatively spliced genes. For example, a stage-associated exon might encode a domain of a transcription factor that binds to a non-AS gene, and thus might regulate the expression of that gene.

Our work presents an important fact: the alternative splicing (AS) patterns of a certain group of exons can well represent the overall transcriptome dynamics of cells. This poses an interesting question to biologists: is it possible to regulate cell differentiation process by changing the AS patterns of certain exons? Previous studies have shown critical roles of AS in cell differentiation: [35] showed that regulating an intricate network of nervous system specific AS promotes neuronal differentiation; [36] found an important role of AS in the specification of mouse embryonic stem cells in differentiation; [37] revealed that AS events regulate pluripotency through the control of critical embryonic stem cell-specific transcriptional programs. It is meaningful to further explore the roles of conserved exons in cell differentiation. Another inspiration is the pivotal role of conserved AS in the exploration of inter-species conservation. [38] found a significant number of apparent nonsense-mediated mRNA decay (NMD) inducing AS isoforms in the conserved AS isoforms of mouse and human, and NMD is an important regulation mechanism for gene expression. [16] showed that some conserved AS events between *Arabidopsis* and rice form a strongly conserved mechanism of post-transcriptional regulation. These studies, together with our work, show that it is valuable to study inter-species conservation from the perspective of conserved AS.

## Conclusions

We have reported a statistical study to find correspondence of *D. melanogaster* and *C. elegans* developmental stages based on alternative splicing characteristics of conserved cassette exons. We construct correspondence map by mapping “associated exons” in pairwise biological samples. Two parallel mapping patterns are observed in the comparison of fly and worm developmental stages. We expect that our study would highlight the importance of inter-species AS conservation, and our identified conserved cassette exons associated with different fly and worm samples would serve as interesting targets for biologists to further explore the conservation of developmental biology.

## Methods

### A Strategy to compare fly and worm developmental stages, tissues and cells based on alternative splicing characteristics of conserved exons

**Fig. 8:**
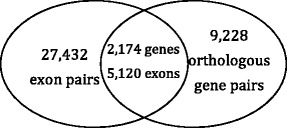
Venn graph showing the relationship between exon pairs and orthologous gene pairs

To compare fly and worm in terms of alternative splicing, we first identify their conserved exons by aligning fly and worm exons using BLAST [39], which has been widely used to predict orthologous genes, such as in TreeFam [40] and Ensembl [41]. In contrast to the orthologous gene prediction for which protein sequence similarity is the major determinant, in our prediction of conserved exons, DNA sequence similarity is the key. We collect fly and worm exons and their sequences from the Ensembl annotation and reference genome (version 66) [42]. We find exons with DNA sequence similarity by an E-value cutoff of 10^-5^ in the reciprocal mapping of fly and worm exons using BLAST, resulting in 27,432 exon pairs (Fig. 8). We define conserved exon pairs by restricting the exons pairs in orthologous gene pairs.

We are interested in the alternative splicing characteristics of the conserved exons across fly and worm developmental stages. Hence, we use the modENCODE RNA-seq data of *D. melanogaster* and *C. elegans* developmental stages to estimate the expression level of two transcripts that only differ by one target exon of interest: “transcript I” including the exon and “transcript S” excluding the exon. The transcript expression levels are estimated by Cufflinks (version 2.2.1) in FPKM (Fragments Per Kilobase of transcript per Million mapped reads) units [43]. By removing the exons that are not alternatively spliced at any stages, tissues or cells (i.e., transcript I or transcript S has zero expression estimate across all stages, tissues or cells), we retain 2148 conserved exon pairs for the rest of our analysis. The conserved exon pairs belong to 1124 orthologous gene pairs: 627 gene pairs have single conserved exon pairs, while 497 gene pairs have multiple conserved exon pairs. To quantify the alternative splicing characteristics of every retained exon, we calculate its inclusion/skipping (I/S) ratio as (expression level of transcript I)/(expression level of transcript S).

To capture the alternative splicing characteristics of every fly (or worm) biological sample, we identify exons whose I/S ratios capture the transcriptome characteristics of a particular sample. Specifically, for developmental stages, we select its *stage-associated exons* whose I/S ratios are relatively high or low at that stage compared with some other fly (or worm) stages. We use the similar approach to select *tissue-/cell-associated exons* as those that are highly included or skipped compared to other tissue/cell samples. This is motivated by the fact that an exon with constant I/S ratio across all biological samples provides little information to differentiate particular samples from others in terms of alternative splicing. We define an exon to be associated with a sample if its *Z*-score (normalized I/S ratio across stages) at that sample is greater than 1.5 or lower than -1.5. This criterion enables us to select the exons that have much higher or lower I/S ratios in a given sample compared to some other samples; in other words, the AS patterns of these selected exons in that sample could be similar with some samples but must be greatly deviated from a few other samples. Hence, the selected stage-associated exons provide a basis for comparing the AS events of different samples. We expect the samples that share more associated exons to be more similar in terms of AS patterns. Using this selection approach, for every fly and worm biological sample, we identify highly included associated exons (with *Z*-scores greater than 1.5), whose numbers range from ~50 to ~1900, and lowly included associated exons (with *Z*-scores lower than -1.5), whose numbers range from ~40 to ~1600.

**Fig. 9:**
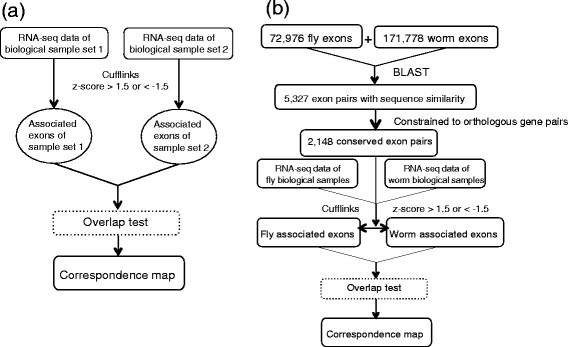
Outline of the strategy to compare biological samples. **a** Outline of the strategy to compare two samples within a species. **b** Outline of the strategy to compare two samples between two species. We define an exon to be associated with a sample if its *Z*-score (normalized I/S ratio across stages) at that sample is greater than 1.5 or lower than -1.5

We compare a pair of biological samples by statistically testing the dependence of their associated exons using an *overlap test*. Specifically, if the two samples are within the same species, we test the significance of the number of associated exons they share; if the two samples are from different species, we test the significance of the number of conserved exon pairs shared in their associated exons. The test is under a null hypothesis that their associated exons are two independent samples from the population, i.e., the highly included exons and lowly included exons from all samples (for within-species comparison) or all the conserved exon pairs (for between-species comparison). The larger the number of associated exons they share or the larger the number of conserved exon pairs existing in their associated exons, the more likely the null hypothesis will be rejected. We define a mapping score of this sample pair as − log_10_ (*p*-value), where the *p*-value is returned by the *overlap test*. Figure 9 illustrates our strategy.

### Finding exon pairs with high sequence similarity between *D. melanogaster* and *C. elegans*

BLAST (version 2.2.31+, blastn program, using “-task blastn” option; [39]) is used to compare the sequence similarity between 72,976 *D. melanogaster* exons and 171,778 *C. elegans* exons. Exons are regarded as conserved if they are in an orthologous gene pair and meet the E-value constraint of 10^-5^ (i.e., E-value < 10^-5^ when taking fly exons as database and worm exons as query, and vice versa.). Reference annotation and sequence are obtained from Ensembl assembly 66 (i.e., BDGP 5.64 for *D. melanogaster* and WS 220 for *C. elegans*). Among the 27,432 pairs of exons found by BLAST, we select those pairs whose exons both have a left neighbor exon and a right neighbor exon in a transcript, also based on the Ensembl annotations.

### Estimating inclusion/skipping ratios of exons

**Fig. 10:**
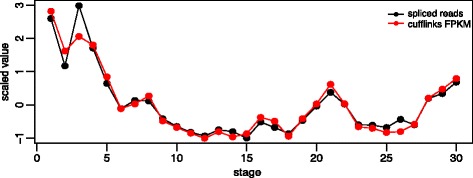
Comparison of the expression estimates given by Cufflinks and naïve counting for the transcript I of a fly exon (chr3R: 5597918-5598871). The naïve counting estimates are normalized by number of total mapped reads. We can see the two trends match almost perfectly with each other

We use Cufflinks (version 2.2.1, supplied with reference annotation, i.e., using “-G” option; [43]) to quantify the inclusion and skipping levels of an exon. To fulfill that aim, we provide a “pseudo” transcript annotation to Cufflinks; that is, for every exon, we construct two transcripts: 1) “transcript S” (skipped): a two-exon-transcript that connects the left neighbor and right neighbor of the exon, and 2) “transcript I” (included): a three-exon-transcript that connects the left neighbor, the exon and the right neighbor together. For every exon, the expression estimates of the two transcripts are returned by Cufflinks in FPKM (fragments per kilobase of transcript per million mapped reads) units. The reason we use this approach is the good computational efficiency of Cufflinks. We confirm that the expression estimates of these two “pseudo” transcripts well indicate the skipping and inclusion levels of the exon. That is, for several individual genes we naïvely count the reads that crossed the relevant exon junctions in the inclusion and skipping cases in every developmental stage. The trends given by Cufflinks (in FPKM unit) and given by naïve counting across all the stages almost perfectly match each other (see Fig. 10 as an example). We are interested in the exons whose inclusion and skipping cases both occur in at least one developmental stage, because this indicates that the exon is an alternatively spliced cassette exon. Hence, we select the exons whose included transcript FPKM and skipped transcript FPKM are both positive for at least one stage. This gives us 5327 cassette exon pairs. We further restrict ourselves to the cassette exon pairs in orthologous gene pairs, resulting in 2148 conserved exon pairs. To quantify the alternative splicing level of an exon, we convert the FPKMs returned by Cufflinks to the following inclusion/skipping (I/S) ratio:

$$ \mathrm{I}/\mathrm{S}\ \mathrm{ratio} = \log \left(\frac{\mathrm{transcript}\ \mathrm{I}\ \mathrm{FPKM}+1}{\mathrm{transcript}\ \mathrm{S}\ \mathrm{FPKM}+1}\right)\ . $$

Hence, every fly and worm exon has an I/S ratio for every developmental stage, tissue and cell.

### Smoothing the trend of inclusion/skipping ratios across developmental stages

Since AS dynamics should have continuous change patterns during development, in order to remove noise and extract the main trend of I/S ratio changes across fly and worm developmental stages, for every exon we smooth its I/S ratios across stages. We use the R function *loess()* (with option “span = 0.3”), which aims at locally fitting a polynomial surface for smoothing. This step is important for the identification of stage-associated exons in next step, because if an exon has a sharp spike change in its I/S ratios at a certain stage, it should not be chosen as an associated exon of that stage, and the smoothing step can help relieve this issue.

### Identification of associated exons

The algorithm to identify associated exons is similar to the identification of associated genes in [11] and [44]. That is, for every fly exon, suppose its I/S ratios in *n* biological samples (i.e. developmental stages, tissue or cells) are *e*_*1*_ *, e*_*2*_ *, …, e*_*n*_. We normalize the ratios as *z*_*1*_ *, z*_*2*_ *, …, z*_*n*_ *,* where ${z}_i = \frac{e_i-\overline{e}}{s}, i=1,\dots, n$ are the normalized *Z*-scores, with $\overline{e} = \frac{1}{n}{\displaystyle {\sum}_{i=1}^n{e}_i}$ and $s = \sqrt{\frac{1}{n-1}{\displaystyle {\sum}_{i=1}^n{\left({e}_i-\overline{e}\right)}^2}}$ as the mean and standard deviation of the *n* I/S ratios. For each fly sample, we would like to select the fly exons whose I/S ratios are relatively high or relatively low compared to other samples. The selection threshold we use in this study is *z*_*i*_ ≥ 1.5 or *z*_*i*_ ≤ -1.5. For worm biological samples, we use the same method and threshold to select associated exons.

### Overlap test for within-species comparison

Given two biological samples of the same species (i.e, *D. melanogaster* or *C. elegans*), we compare them by testing the dependence of their associated exons, denoted by exon sets *A* and *B*. We consider each cassette exon in two types of AS status (a highly included exon and a lowly included exon), resulting in a doubled number of cassette exons in the population. We consider the associated exon sets *A* and *B* as two samples drawn from the population, and design an *overlap* *test* for the common exons in *A* and *B*. The null hypothesis is that *A* and *B* are two independent samples from the same population; the alternative hypothesis is that *A* and *B* are dependent. The test statistic is the number of associated exons (required to be in the same AS status, i.e., highly included or lowly included) shared by *A* and *B*, denoted by |*A* ∩ *B*|. Given the sizes of *A* and *B*, the larger the test statistic is, the higher the likelihood is that the null hypothesis will be rejected. We calculate the *p*-value of the statistic as the right tail probability under the null hypothesis

$$ p={\displaystyle {\sum}_{i=\left| A{\displaystyle \cap } B\right|}^{min\left(\left| A\right|,\left| B\right|\right)}\frac{\left(\begin{array}{c}\hfill n\hfill \\ {}\hfill i\hfill \end{array}\right)\left(\begin{array}{c}\hfill n- i\hfill \\ {}\hfill \left| A\right|- i\hfill \end{array}\right)\left(\begin{array}{c}\hfill n-\left| A\right|\hfill \\ {}\hfill \left| B\right|- i\hfill \end{array}\right)}{\left(\begin{array}{c}\hfill n\hfill \\ {}\hfill \left| A\right|\hfill \end{array}\right)\left(\begin{array}{c}\hfill n\hfill \\ {}\hfill \left| B\right|\hfill \end{array}\right)}}, $$

where *n* are twice the total number of cassette exons and |*A*| and |*B*| are the numbers of associated exons in the sets *A* and *B*. Since a smaller *p*-value indicates stronger dependency of two samples, we convert the *p*-value to a mapping score − log_10_ (*p*-value). Then for the *n* biological samples of fly or worm, we obtain an *n* × *n* matrix of mapping scores.

### Overlap test for between-species comparison

Given two biological samples of different species, we compare them by testing the conservation of their associated exons. We use the aforementioned 2148 conserved exon pairs. Since there are two types of associated exons, with *z*_*i*_ relatively high for highly included exons or relatively low for lowly included exons, we separate each conserved exon pairs into two pairs to represent the two types, resulting in a two-column array of 4296 rows (*h* for highly included, and *l* for lowly included):

$$ \begin{array}{r}\hfill \mathrm{fly}\kern0.5em \mathrm{exon}\\ {}\hfill {f}_{h,1}\\ {}\hfill \dots \\ {}\hfill {f}_{h,2148}\\ {}\hfill {f}_{l,1}\\ {}\hfill \dots \\ {}\hfill {f}_{l,2148}\end{array}\kern1.5em \begin{array}{c}\hfill \kern1em \hfill \\ {}\hfill \kern0.5em \leftrightarrow \hfill \\ {}\hfill \kern1em \hfill \\ {}\hfill \kern0.5em \leftrightarrow \hfill \\ {}\hfill \kern0.5em \leftrightarrow \hfill \\ {}\hfill \kern1em \hfill \\ {}\hfill \kern0.5em \leftrightarrow \hfill \end{array}\kern1.5em \begin{array}{l}\mathrm{worm}\kern0.5em \mathrm{exon}\hfill \\ {}{w}_{h,1}\hfill \\ {}\dots \hfill \\ {}{w}_{h,2148}\hfill \\ {}{w}_{l,1}\hfill \\ {}\dots \hfill \\ {}{w}_{l,2148}\hfill \end{array} $$

Suppose *F* (and *W*) are the fly (and worm) associated exon sets contained in these conserved fly-worm exon pairs. *F* and *W* contain no repetitive exons, while the aforementioned 4296 rows contain one-to-many, many-to-one and many-to-many conserved exon pairs. Hence, we define *F*′ = {*f*_*h*,*i*_ : if *f*_*h*,*i*_ ∈ *F*, *i* = 1, …, 2148} ∪ {*f*_*l*,*i*_ : if *f*_*l*,*i*_ ∈ *F*, *i* = 1, …, 2148} and *W*′ = {*w*_*h*,*i*_ : if *w*_*h*,*i*_ ∈ *W*, *i* = 1, …, 2148} ∪ {*w*_*l*,*i*_ : if *w*_*l*,*i*_ ∈ *W*, *i* = 1, …, 2148}. Then we regard *F*′ as a sample from {*f*_*h*,1_, …, *f*_*h*,2148_, *f*_*l*,1_, …, *f*_*l*,2148_} and *W*′ as a sample from {*w*_*h*,1_, …, *w*_*h*,2148_, *w*_*l*,1_, …, *w*_*l*,2148_}. Because of the one-to-one relationship between {*f*_*h*,1_, …, *f*_*h*,2148_, *f*_*l*,1_, …, *f*_*l*,2148_} and {*w*_*h*,1_, …, *w*_*h*,2148_, *w*_*l*,1_, …, *w*_*l*,2148_}, we can consider *F*′ and *W*′ as two samples from the same population.

In our overlap test, the null hypothesis is that *F*′ and *W*′ are independent samples from the population; the alternative hypothesis is that *F*′ and *W*′ are dependent samples. This becomes an *overlap* test, and the test statistic is the number of conserved exon pairs existing between *F*′ and *W*′, defined as *T*. The larger the statistic is, the higher the likelihood is that the null hypothesis will be rejected. The *p*-value of the test statistic is calculated as

$$ p={\displaystyle {\sum}_{i= T}^{min\left(\left|{F}^{\prime}\right|,\left|{W}^{\prime}\right|\right)}\frac{\left(\begin{array}{c}\hfill 4296\hfill \\ {}\hfill i\hfill \end{array}\right)\left(\begin{array}{c}\hfill 4296- i\hfill \\ {}\hfill \left|{F}^{\prime}\right|- i\hfill \end{array}\right)\left(\begin{array}{c}\hfill 4296-\left|{F}^{\prime}\right|\hfill \\ {}\hfill \left|{W}^{\prime}\right|- i\hfill \end{array}\right)}{\left(\begin{array}{c}\hfill 4296\hfill \\ {}\hfill \left|{F}^{\prime}\right|\hfill \end{array}\right)\left(\begin{array}{c}\hfill 4296\hfill \\ {}\hfill \left|{W}^{\prime}\right|\hfill \end{array}\right)},} $$

where |*F*′| and |*W*′| are the numbers of elements in sets *F*′ and *W*′.

Since a smaller *p*-value indicates stronger dependency of two samples, we convert the *p*-value into a mapping score = −log_10_ (*p*-value). Then for *n* fly samples and *m* worm samples, we obtained an *n* × *m* matrix of mapping scores, which we illustrate as a correspondence map.

## Additional files

Additional file 1: Illustration of RNA-seq datasets. Illustration of RNA-seq datasets of fly and worm from modEncode. (PDF 1020 kb)
Additional file 2: Heat maps of GO terms. Summary of the enriched GO terms in fly and worm developmental stages. (PDF 308 kb)
Additional file 3: Heat maps of KEGG. Summary of the enrichment of KEGG pathways in fly and worm developmental stages. (PDF 229 kb)
Additional file 4: Functional annotations of protein domains. Conserved functional annotations of protein domains encoded by conserved cassette exon pairs. (XLSX 146 kb)
Additional file 5: Exon pairs. Conserved exon pairs and other exon pairs with high DNA-seq similarity. (XLSX 446 kb)
Additional file 6: Stage-associated exons. Associated exons of fly and worm developmental stages. (XLSX 641 kb)
Additional file 7: Tissue/cell-associated exons. Associated exons of fly and worm tissue/cells. (XLSX 464 kb)
Additional file 8: Blast score and inclusion/skipping ratio of exon pairs. Alignment score reported by BLAST and inclusion/skipping ratio calculated using FPKM reported by Cufflinks of exon pairs with high DNA-seq similarity. (XLSX 6077 kb)

## Abbreviations

AS: Alternative splicing
BP: Biological process
fly: *Drosophila melanogaster*
GO: Gene ontology
I/S: Inclusion/skipping
KEGG: Kyoto Encyclopedia of Genes and Genomes
modENCODE: The Model Organism ENCyclopedia of DNA Elements
worm: *Caenorhabditis elegans*
