# Supplementary material for: Correspondence of D. melanogaster and C. elegans developmental stages revealed by alternative splicing characteristics of conserved exons
Source: BMC Genomics. 2017 Mar 16;18:234. doi: 10.1186/s12864-017-3600-2 (PMC5353869; doi:10.1186/s12864-017-3600-2)
Supplement: Additional file 1: — Illustration of RNA-seq datasets. Illustration of RNA-seq datasets of fly and worm from modEncode. (PDF 1020 kb) [file 12864_2017_3600_MOESM1_ESM.pdf]

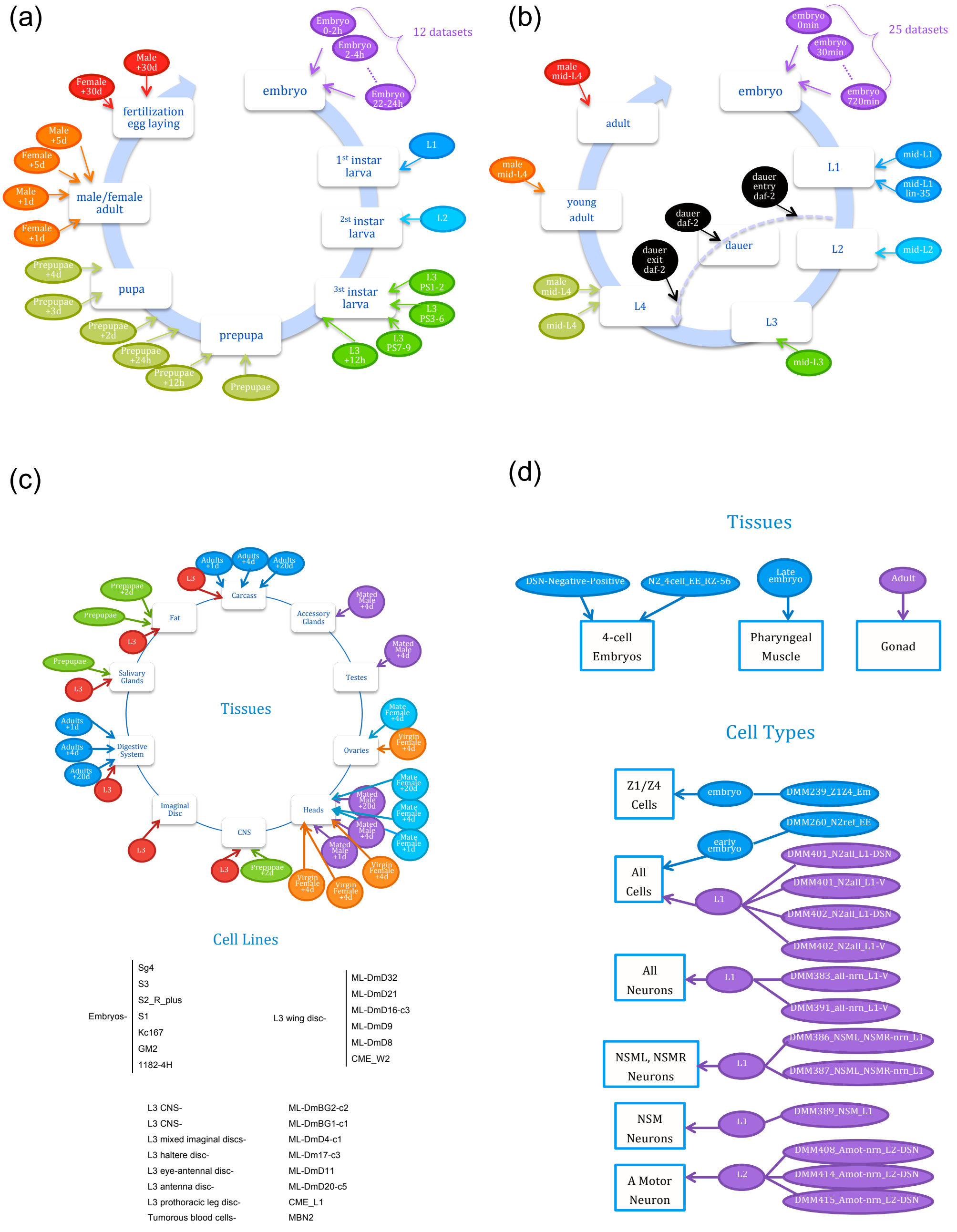

Figure S1. Life cycles and modENCODE RNA-seq data sets of *D. melanogaster* and *C. elegans*. This figure has been adapted from Li et. al 2014. (a) modENCODE RNA-seq data sets of 30 *D. melanogaster* developmental stages. (b) modENCODE RNA-seq data sets of 36 *C. elegans* developmental stages. (c) modEncode RNA-seq data sets of 29 tissues and 21 cell lines in *D. melanogaster*. (d) modEncode RNA-seq data sets of 4 tissues and 14 dissected cell. in *C. elegans*.
