# Supplementary material for: Correspondence of D. melanogaster and C. elegans developmental stages revealed by alternative splicing characteristics of conserved exons
Source: BMC Genomics. 2017 Mar 16;18:234. doi: 10.1186/s12864-017-3600-2 (PMC5353869; doi:10.1186/s12864-017-3600-2)
Supplement: Additional file 2: — Heat maps of GO terms. Summary of the enriched GO terms in fly and worm developmental stages. (PDF 308 kb) [file 12864_2017_3600_MOESM2_ESM.pdf]

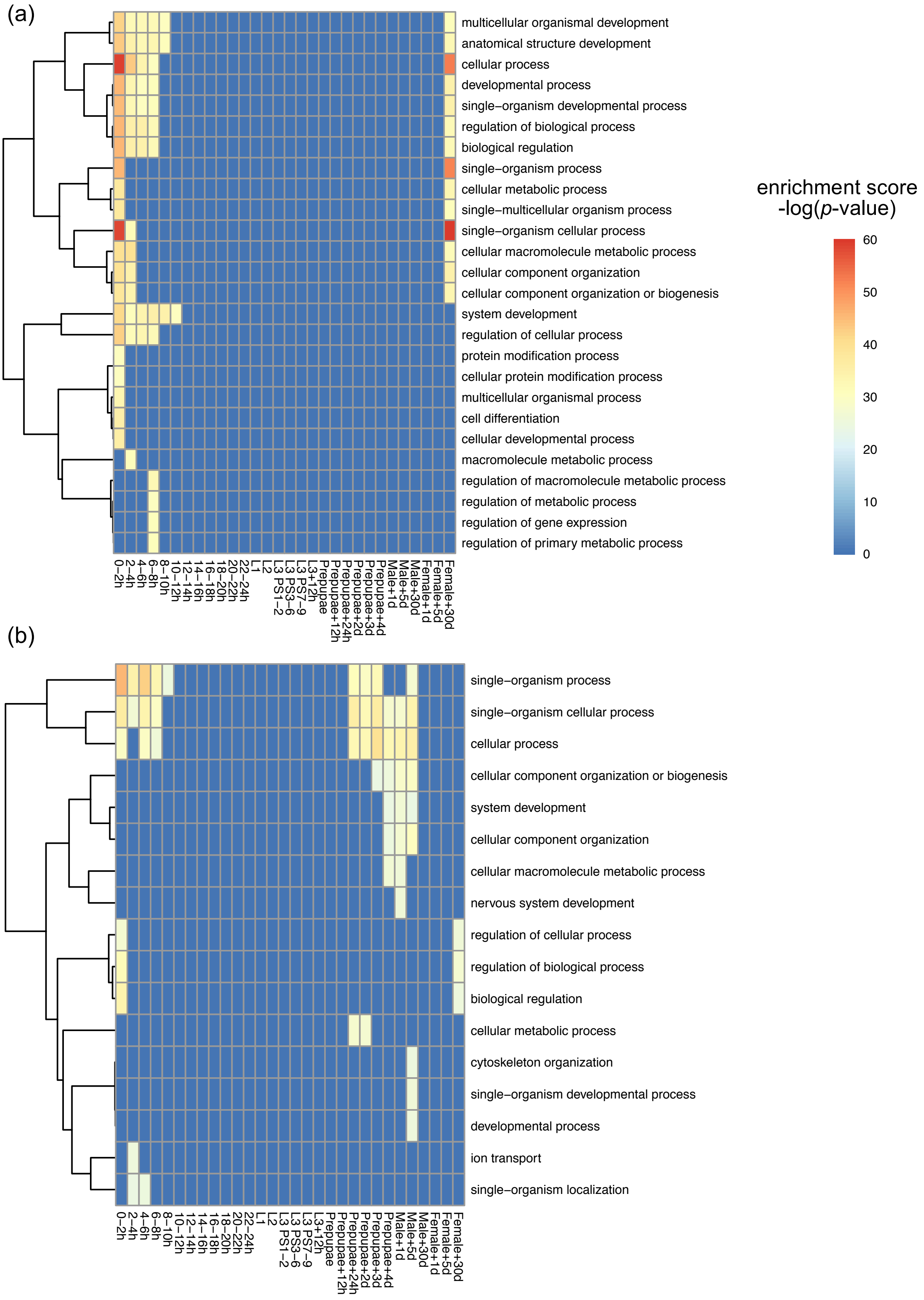


Figure S2. Biological process (BP) gene ontology (GO) analysis of (a) highly included fly stage-associated exons and (b) lowly included fly stage-associated exons. The enrichment scores shown on the heatmaps are $-\log_{10}(p-\text{value})$ from hypergeometric test. Here we list the GO terms whose $p-\text{values}$ are at least less than $10^{-30}$ in one stage.

(a)

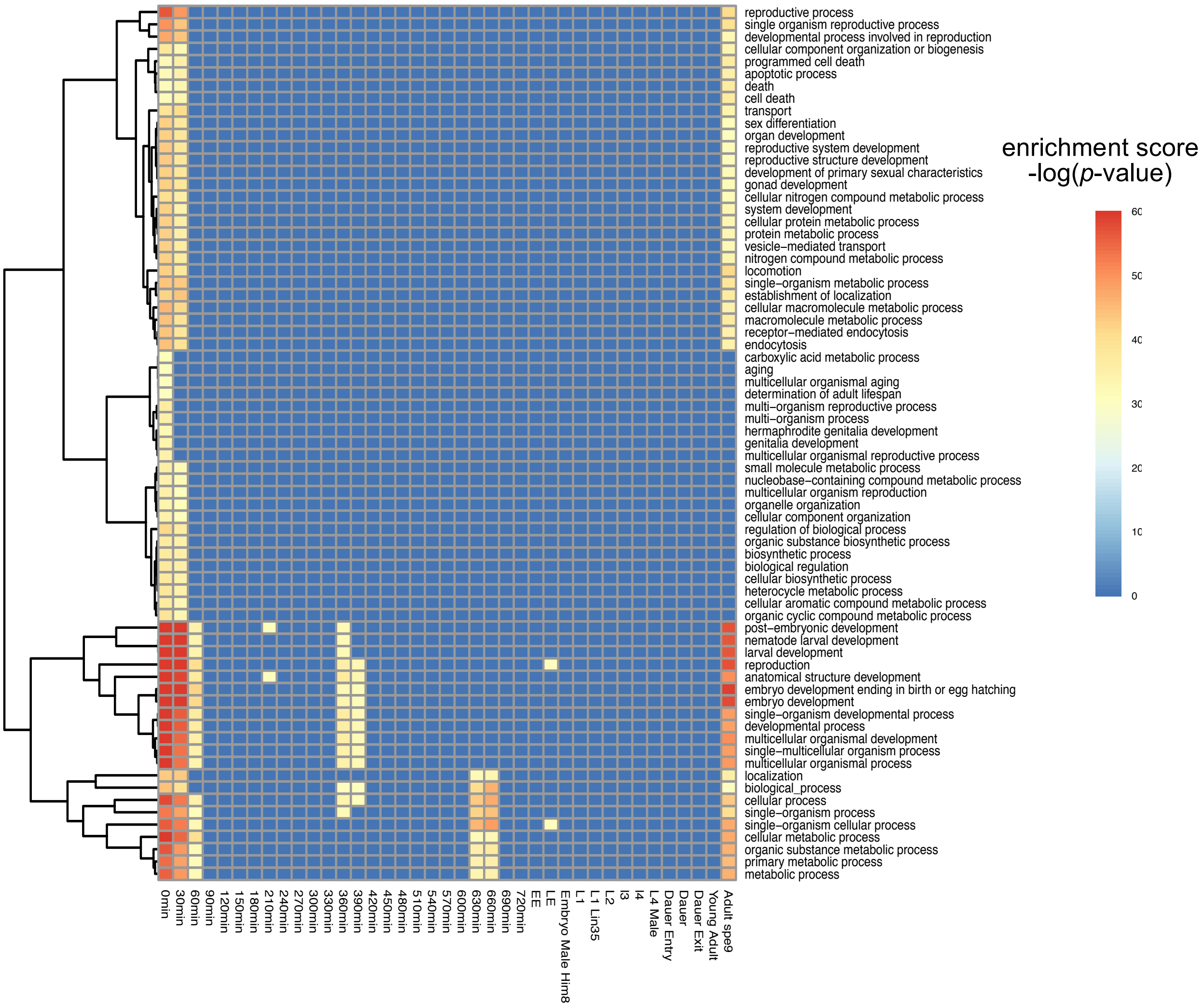


Figure S3. (Continued on next page)

(b)

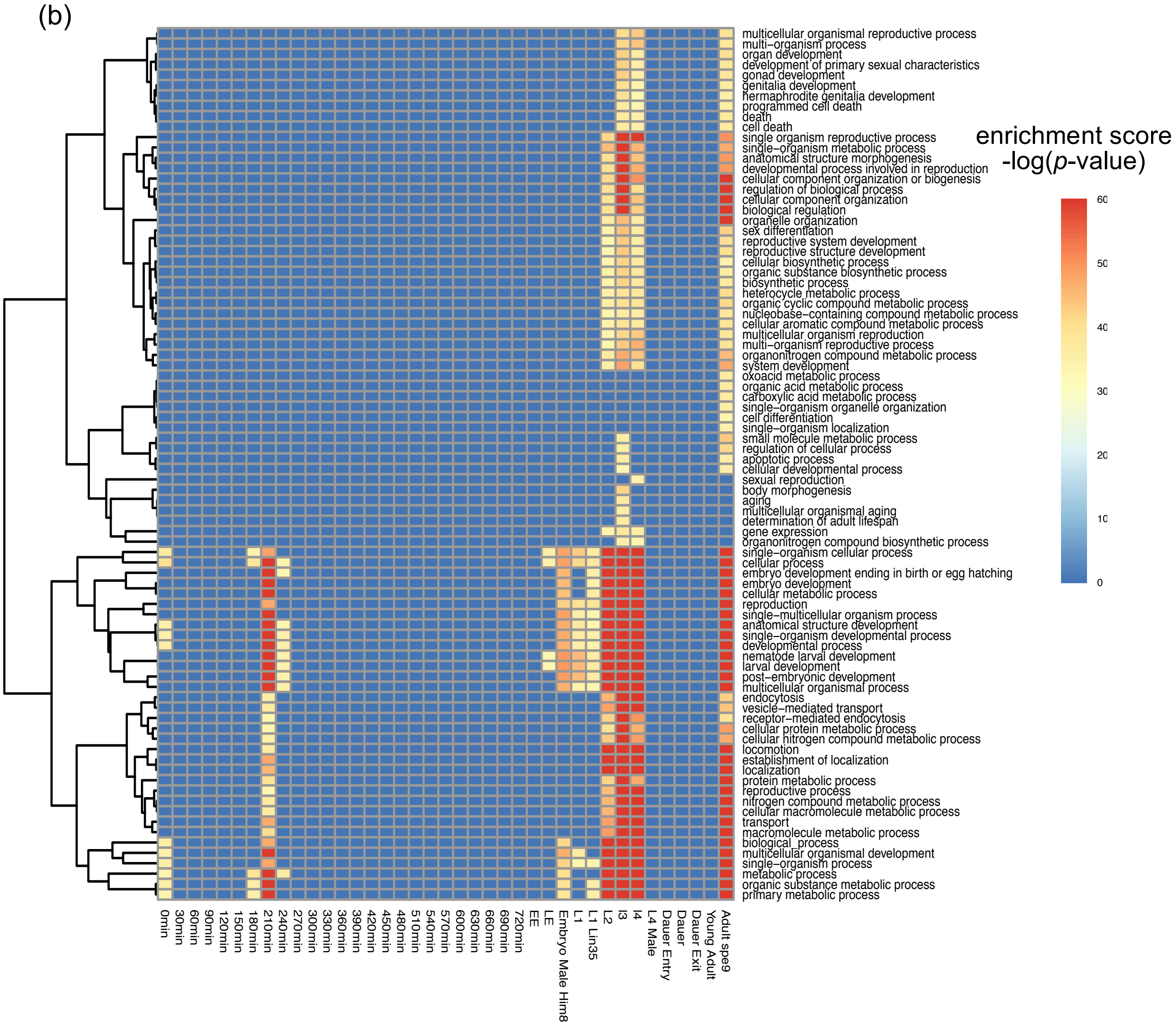


Figure S3. Biological process (BP) gene ontology (GO) analysis of (a) highly included worm stage-associated exons and (b) lowly included worm stage-associated exons. The enrichment scores shown on the heatmaps are $-\log(P-value)$ from the hypergeometric tests. Here list GO terms whose $P-value$ is at least less than $10^{-30}$ in one stage.
