# Supplementary material for: Correspondence of D. melanogaster and C. elegans developmental stages revealed by alternative splicing characteristics of conserved exons
Source: BMC Genomics. 2017 Mar 16;18:234. doi: 10.1186/s12864-017-3600-2 (PMC5353869; doi:10.1186/s12864-017-3600-2)
Supplement: Additional file 3: — Heat maps of KEGG. Summary of the enrichment of KEGG pathways in fly and worm developmental stages. (PDF 229 kb) [file 12864_2017_3600_MOESM3_ESM.pdf]

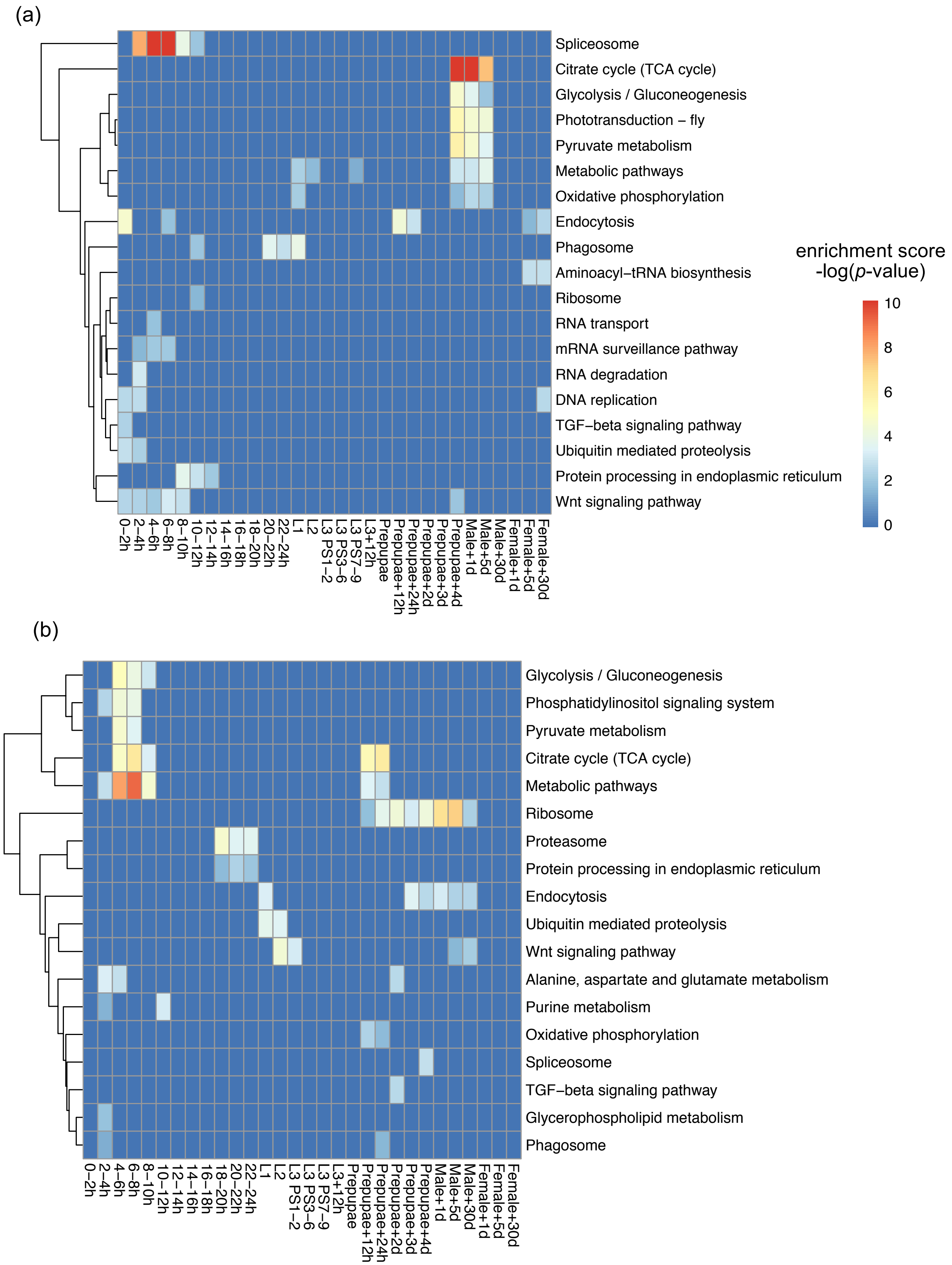

Figure S4. Kyoto Encyclopedia of Genes and Genomes (KEGG) enrichment analysis of (a) fly highly included stage-associated exons and (b) fly lowly included stage-associated exons. The enrichment scores shown on the heatmaps are $-\log_{10}(p-\text{value})$ from hypergeometric test. Here we list the KEGG pathways whose $p-\text{values}$ are at least less than 10^{-7} at one stage.

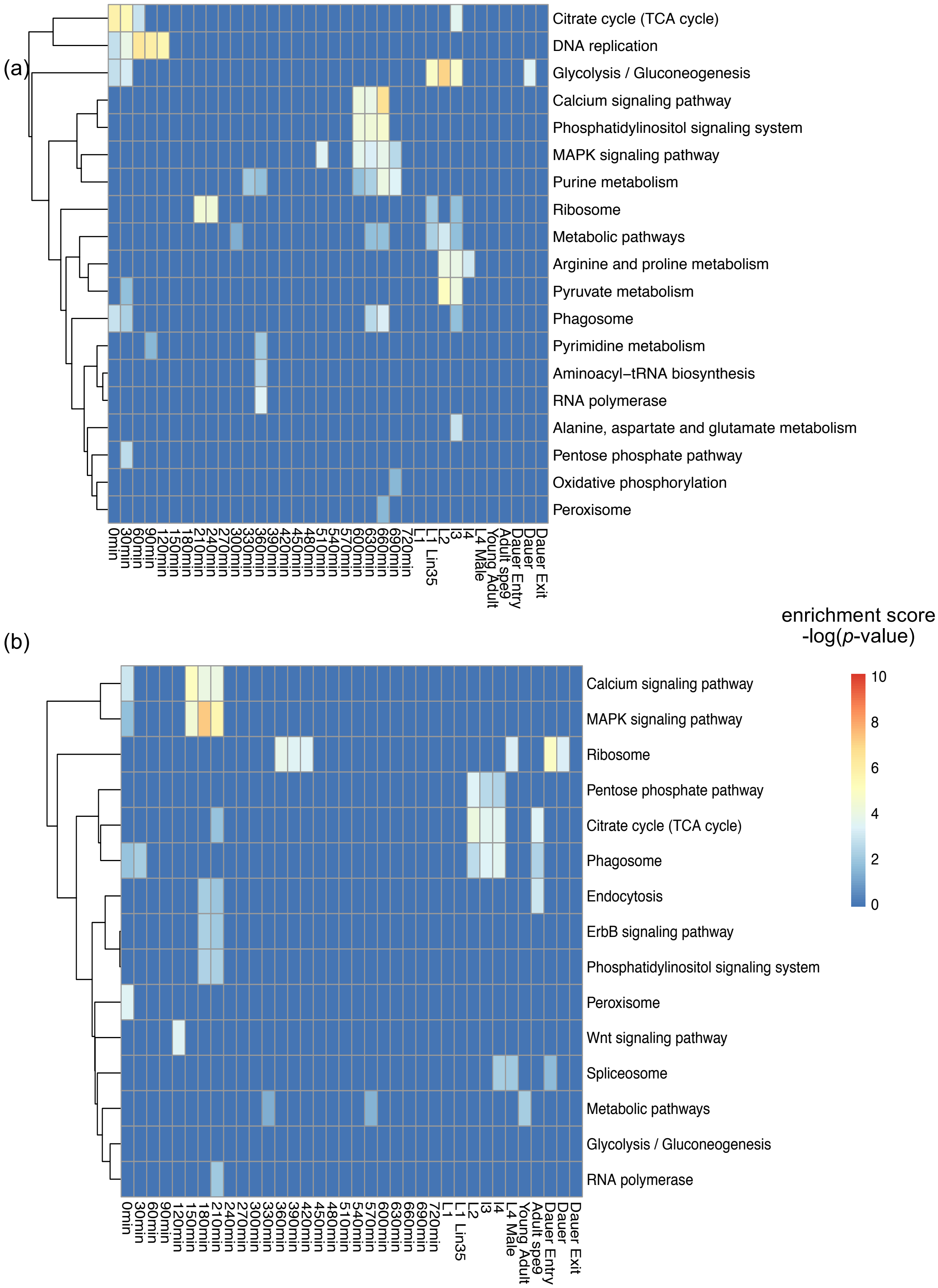

Figure S5. Kyoto Encyclopedia of Genes and Genomes (KEGG) enrichment analysis of (a) worm highly included stage-associated exons and (b) worm lowly included stage-associated exons. The enrichment scores shown on the heatmaps are $-\log_{10}(p-\text{value})$ from hypergeometric test. Here we list the KEGG pathways whose $p-\text{values}$ are at least less than 10^{-7} at one stage.
